# A Virus in American Blackcurrant (*Ribes americanum*) with Distinct Genome Features Reshapes Classification in the *Tymovirales*

**DOI:** 10.3390/v10080406

**Published:** 2018-08-03

**Authors:** Thanuja Thekke-Veetil, Thien Ho, Joseph D. Postman, Robert R. Martin, Ioannis E. Tzanetakis

**Affiliations:** 1 Department of Plant Pathology, Division of Agriculture, University of Arkansas System, Fayetteville, AR 72701, USA; thanujatv@gmail.com (T.T.-V.); thienxho@gmail.com (T.H.); 2 National Clonal Germplasm Repository, United States Department of Agriculture, Corvallis, OR 97333, USA; joseph.postman@ars.usda.gov; 3 Horticultural Crops Research Unit, United States Department of Agriculture, Corvallis, OR 97331, USA; bob.martin@ars.usda.gov

**Keywords:** *Betaflexiviridae*, blackcurrant, Ribes americanum virus A, characterization, detection

## Abstract

A novel virus with distinct genome features was discovered by high throughput sequencing in a symptomatic blackcurrant plant. The virus, tentatively named Ribes americanum virus A (RAVA), has distinct genome organization and molecular features bridging genera in the order *Tymovirales*. The genome consists of 7106 nucleotides excluding the poly(A) tail. Five open reading frames were identified, with the first encoding a putative viral replicase with methyl transferase (MTR), AlkB, helicase, and RNA dependent RNA polymerase (RdRp) domains. The genome organization downstream of the replicase resembles that of members of the order *Tymovirales* with an unconventional triple gene block (TGB) movement protein arrangement with none of the other four putative proteins exhibiting significant homology to viral proteins. Phylogenetic analysis using replicase conserved motifs loosely placed RAVA within the *Betaflexiviridae*. Data strongly suggest that RAVA is a novel virus that should be classified as a species in a new genus in the *Betaflexiviridae* or a new family within the order *Tymovirales*.

## 1. Introduction

**Figure 1:**
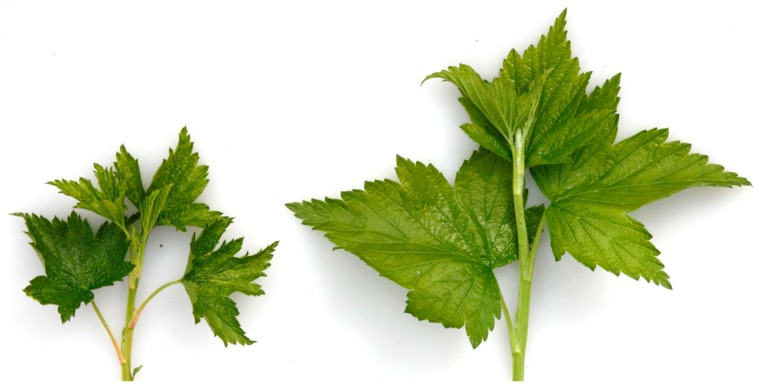
Symptoms observed on American blackcurrant infected with Ribes americanum virus A. Infected *Ribes americanum* cultivar Gall (PI 617879, **left**) showing ragged leaf margins and crinkled leaf surface compared to healthy plant (**right**).

Technological developments in high throughput sequencing (HTS) have resulted in the rapid discovery and characterization of several novel plant viruses [1,2,3,4]. The USDA National Clonal Germplasm Repository (NCGR) maintains and distributes various accessions of specialty fruit and nut crop species from around the world [5]. This includes currant (*Ribes* spp.), a berry known for its potential health effects [6]. This study was initiated when an American blackcurrant maintained in the USDA-NCGR, Corvallis, Oregon (*Ribes americanum*; PI 617879) showed virus-like symptoms (Figure 1). The plant was infected with two viruses [3]: A waikavirus [7] and an undescribed virus in the order *Tymovirales*.

The order *Tymovirales* includes five families, namely *Alphaflexiviridae*, *Betaflexiviridae*, *Gammaflexiviridae*, *Deltaflexiviridae*, and *Tymoviridae* [8]. Members of this group are non-enveloped, flexuous, filamentous viruses primarily infecting plants, and have genomes resembling eukaryotic mRNAs [9]. This manuscript describes the characterization of a novel virus tentatively named as Ribes americanum virus A (RAVA). The evolutionary relationship of RAVA with other known members of the order *Tymovirales* indicated that RAVA is a virus with unique genome features, possibly representing a new genus in the *Betaflexiviridae* family, or being the type representative of a yet to be described family. Discovery of this novel virus adds information to the current knowledge on this diverse group of viruses, and expands the number of virus species reported in the order.

## 2. Materials and Methods

Double-stranded RNAs were extracted from 20 g of symptomatic leaves from currant accession PI 617879 (Figure 1) and subjected to a degenerate oligonucleotide-primed reverse transcription-PCR (DOP RT-PCR) followed by HTS and sequence assembly as described [10]. The FirstChoice^®^ RLM-RACE Kit (ThermoFisher Scientific, Waltham, MA, USA) was used to obtain the 5′ terminus. The 5′ and 3′ RACE-RT-PCRs were performed using virus-specific primers and programs described in Table S1. For RACE and confirmation of genome regions downstream of ORF 1 total nucleic acids were extracted as described [3]. For all amplification reactions, Takara LA Taq polymerase (Takara Bio USA Inc, Mountain View, CA, USA) was used according to the manufacturer’s instructions. All PCR products were sequenced for at least three-fold coverage of the regions. The complete nucleotide sequence of RAVA was deposited in GenBank under accession number MF166685.

The genome organization, putative ORFs, and protein sequences were derived using the NCBI ORF Finder [11]. RNA binding amino acid (aa) residues present in RAVA proteins were identified using Pprint (Prediction of Protein RNA-Interaction; [12]) and transmembrane domains identified using TMHMM Server v. 2.0 [13]. The secondary structures of the proteins were predicted by PSI-Pred, I-TASSER [14,15], and nuclear localization signals identified with cNLS Mapper [16]. The protein sequences of members belonging to the families in the order *Tymovirales* were obtained from GenBank (Table 1) and aligned with the virus putative proteins using ClustalW on Bioedit [17]. Phylogenetic analyses were performed using the maximum likelihood method with 1000 bootstrap replicates on MEGA v. 7 [18] and phylogenetic trees displayed using Treeview in MEGA v. 7.

Tissue, tested positive for the virus by RT-PCR, was examined by transmission electron microscopy as follows: Leaf and root tissues were either thin sectioned using a microtome or macerated on a glass slide in water. Sap was applied to a Formvar coated copper EM grid and incubated for 1 min at room temperature. The excess liquid was drawn off with filter paper and samples stained by adding 4 μL of 2% stain for 1 min at room temp before the excess liquid was drawn off using filter paper. For thin sectioning, material was cut into 2 mm sections and fixed in modified Karnovsky fixative overnight at 4 °C. After fixation, the samples were rinsed in 0.1 M sodium cacodylate buffer for 10 min followed by post fixation in 1.5% potassium ferrocyanide and 2% osmium tetroxide in water. Staining was followed by incubations with uranyl acetate overnight and lead aspartate for 30 min at 60 °C. Samples were then dehydrated in acetone (10%, 30%, 50%, 70%, 90%, 95%, 100%) for 10 min each and infiltrated with Araldite resin. Ultrathin sections were done using an RMC PowerTome PC microtome. Grids were stained with either uranyl acetate or phosphotungstic acid and examined in an FEI Titan ChemiSTEM TEM.

## 3. Results

**Figure 2:**
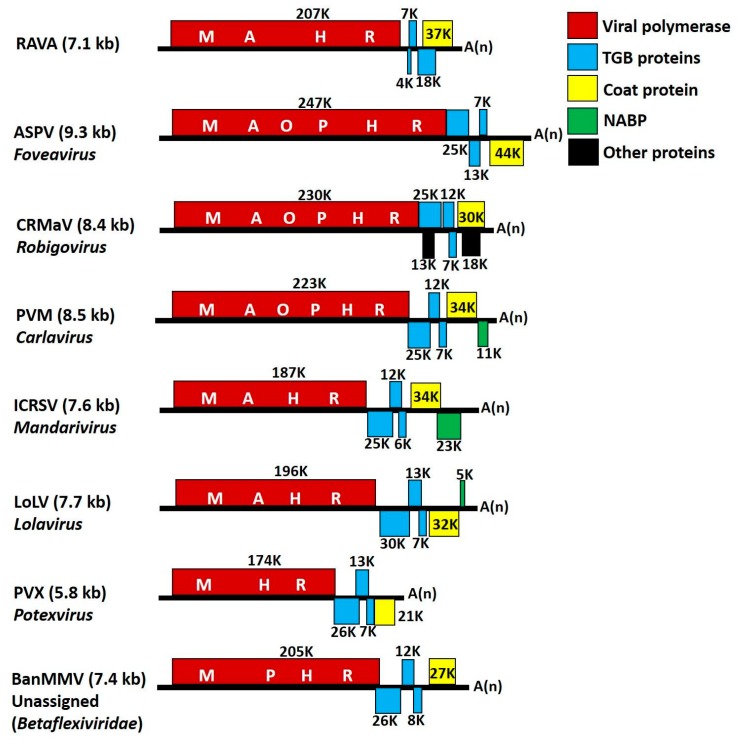
Genome organization of Ribes americanum virus A (RAVA) in comparison to the genomes of members of *Tymovirales* with triple gene block (TGB) movement proteins. M—methyltransferase, A—AlkB, O—OTu-like peptidase, P—papain-like protease, H—helicase, and R—RNA-dependent RNA polymerase. Boxes represent open reading frames. Size of the proteins encoded in the ORFs is indicated. Abbreviations: ASPV—apple stem pitting virus, CRMaV—cherry rusty mottle associated virus, PVM—potato virus M, ICRSV—Indian citrus ringspot virus, LoLV—lolium latent virus, PVX—potato virus X, BanMMV—banana mild mosaic virus.

The genome of RAVA is 7106 nucleotides (nt), excluding the poly-A tail and begins with the pentanucleotide motif GAAAA_1–5_. The genome encodes five ORFs with untranslated regions (UTRs) of 140 and 172 nt for the 5′ and 3′ termini, respectively (Figure 2). 

ORF 1 encodes a putative protein of 207 K (p1; 1826 aa), presumably involved in virus replication as it contains domains of methyl transferase (MTR; aa 44–334; Cdd: pfam01660), AlkB homologue of 2OG-Fe(II) oxygenase (aa 712–806; pfam13532), helicase (HEL; aa 1031–1266; pfam01443), and RNA dependent RNA polymerase (RdRp; aa 1526–1732; pfam00978). The polyprotein contains five nuclear localization signals; one monopartite signal, AVRKRLRFA_1436-44_ and four bipartite signals; FAKCRQLDPENLLLSEALLVNDLIKWLRE_328-65_, KIRLGSKDRVIGSCKDWTTKVIKISKG_913-39_, GCGKTKPLMDLILKSNDNILILVPRKRLGDS_1034-64_, and PRKRLGDSWTSKMGHKKNVRV_1057-78_. The entire protein shares low aa identities with replicases of other members of *Tymovirales* showing the highest identity (23%) to the orthologs in members of the genus *Chordovirus* (carrot Ch virus 1 and 2; CtChV-1 and CtChV-2). The sequence identity of the conserved domains of RdRp ranges from 21–57%; the highest being with members of *Betaflexiviridae* (*Trichovirus* and *Prunevirus*; 55–57%) and the lowest with members of the *Tymoviridae*, *Alpha*-, G*amma*- and *Deltaflexiviridae* (21–32%) (data not shown). The MTR domain also showed higher identity to members of the *Betaflexiviridae* (24–33%) compared to the other families in *Tymovirales*.

Predicted products of ORFs 2–5 showed no significant similarity to viral proteins, and for this reason, HTS products were verified by Sanger sequencing performed on RT-PCR products of viral dsRNA as well as total nucleic acids extracted to eliminate the possibility of contamination (Table S1). ORF 2, which is separated from ORF 1 by an intergenic region of 49 nt encodes a putative peptide of 4 K (p2, 39 aa) with a transmembrane domain (aa_13-35_). ORF 3 partially overlaps ORF 2 and encodes a peptide of 60 aa (p3) with a calculated molecular mass of 7 K with two predicted transmembrane domains at the N and C termini (aa_7-24,34-56_). The secondary structures of the transmembrane regions contain one and two α-helixes respectively (Figure S1). In p2 the exposed hydrophilic regions were positively charged containing four Lys residues at the N terminal and two Arg residues at the C terminal. Both hydrophilic and hydrophobic regions contained nine aa residues predicted to interact with RNA (Table 2). P3 contains only two RNA interacting aa residues and their significance in RNA binding is to be determined (Table 2). 

Separated by an intergenic region of five nt, the fourth ORF encodes a protein of 159 aa with molecular mass of 18K (p4) (Figure 2). This putative protein has marginal identity (24% identity, blossom 45) with a bacterial superfamily 1 (SF1) ATP-dependent DNA helicase domain. The predicted secondary structure (Figure S1) of the protein contains coils, α-helixes, and β-sheets with 60% of the aa residues predicted to interact with RNA (Table 2). These aa appeared as long stretches of primarily positively charged residues (Lys and Arg) which were located in the coils present in the N- and C-termini as well as in the α-helixes. The central region of the protein contained the longest stretch (50 RNA interacting aa) in two α-helixes, which was highly rich in Lys and Arg (30%) in a region mapped to the helicase domain (Table 2).

ORF 5 encodes a putative 37 K (p5, 321 aa) protein of unknown function speculated to be the capsid protein (CP) of the virus. This protein contains several α-helixes and coils and a single β-sheet (Figure S1). Among several RNA binding residues throughout the protein (Table 2), a stretch of 15 residues was predicted in the second and third α-helixes from the N-terminal containing seven Arg residues. The putative CP also has two aa residues (Glu_280,283_) predicted to be involved in ligand binding with calcium (Ca) (I-TASSER; [15]).

**Figure 3:**
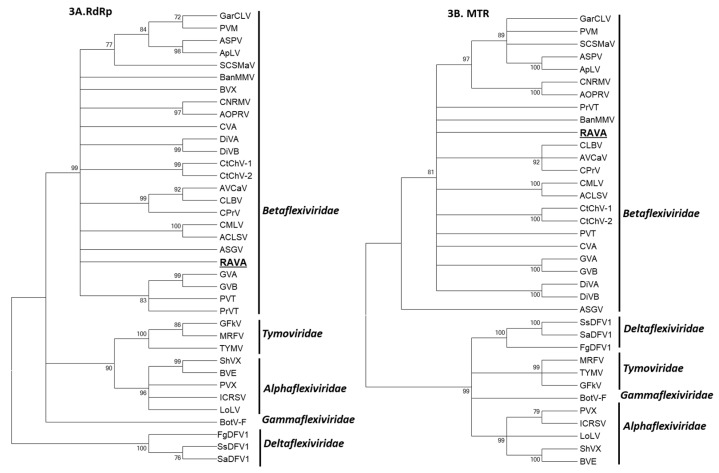
Phylogenetic relationship of Ribes americanum virus A (RAVA) with members of the order *Tymovirales*. Phylogenetic analysis was performed using the conserved domains of RNA-dependent RNA polymerase (RdRp; 3**A**) and methyl transferase (MTR; 3**B**). The trees were generated by the maximum likelihood method using MEGA 7 and bootstrap values (indicated for each branch node) were estimated using 1000 bootstrap replicates. The branches with bootstrap values less than 70% are collapsed. The details of virus isolates used for the studies are provided in Table 1.

The phylogenetic analysis using the conserved domains of the RdRp and MTR (conserved in the alphavirus-like SF [19]) loosely placed RAVA with members of *Betaflexiviridae* (Figure 3A,B), with clustering only supported by low bootstrap values. The analysis based on p4 and CP with the putative orthologs yielded unreliable phylogeny due to extremely low bootstrap values, and is not presented here. 

Tissue tested positive for the virus by RT-PCR was used in electron microscopy. No virus-like particles were identified, nor were there any structures that could belong to an endophyte, a potential host of the virus (data not shown).

## 4. Discussion

RAVA has features bridging genera in both the *Alphaflexiviridae* and *Betaflexiviridae* families. Because of the unique genome organization, we employed electron microscopy on tissue that was tested positive for the virus to determine whether a bacterial or fungal endophyte may be the cryptic host of the virus. In the absence of any structures other than those associated with the plant cell, we determined that RAVA is a plant virus. The genome initiated with the pentanucleotide sequence (GAAAA_1-5_) that is conserved in *Alpha*- and *Betaflexiviridae* [20,21,22,23] and thought to be essential for positive-strand RNA synthesis and protein translation [24,25]. The genome encodes five ORFs with resemblance to the genomes of members with TGB movement proteins, such as *Potexvirus*, *Allexivirus*, *Lolavirus*, *Mandarivirus* (all belonging to *Alphaflexiviridae*) and *Foveavirus*, *Robigovirus*, *Carlavirus*, and the unassigned members (in the *Betaflexiviridae*) (Figure 2). The replicase size is characteristic of a ‘carlavirus-like replicase’ (>195 K), when compared to the ‘potex-like’ (<195 K) [9,26]. This protein contains conserved domains of MTR, HEL, AlkB, and RdRp domains that are present in members of both families. The RAVA replicase showed the highest aa identity to its conserved orthologous domains (MTR and RdRp) of members of the *Betaflexiviridae*. 

Despite the similarity in the 5′ part of the genome coding for the replicase, the RAVA genome differed significantly from genomes of members of the *Alphaflexiviridae* and *Betaflexiviridae* (Figure 2). First and foremost, the putative RAVA MP (p2–p4) and CP do not share any detectable similarity to any known viral proteins. In general, TGB movement proteins are of two types: ‘Hordei-like’ (rod shaped viruses of *Hordeivirus*, *Benyvirus*, *Pomovirus*, and *Pecluvirus*), and ‘potex-like’ (members of *Tymovirales*), and are usually overlapping [27], with the exception of TGBps reported in the genus *Robigovirus* and an unassigned member, sugarcane striate mosaic-associated virus, in which only two, TGBp 2 and TGBp 3, are overlapping. 

The organization of TGB ORFs is highly conserved among plant viruses [27,28]. Potex-like TGB proteins occur in the order of a bigger sized protein, TGBp 1 (~25 K) followed by smaller proteins, TGBp 2 and TGBp 3, (~12 K and ~7 K respectively; Figure 2) in which TGBp 2 has two and TGBp 3 has single transmembrane domains bordered by charged residues. TGBp 2 and TGBp 3 are membrane proteins and localize on endomembranes and cell walls [26,27]. TGBp 1 of these viruses usually harbors an NTP binding helicase domain of SFI and has ATPase, RNA binding, and RNA helicase activity, and is believed to increase the size exclusion limit of plasmodesmata [26]. Based on protein size and characteristics, RAVA seems to have potex-like TGB proteins, however the arrangement of the putative TGB ORFs (ORFs 2–4) is reversed. The RNA binding site of potexviral TGBp1 conserved positively charged residue (Arg), which is essential for interaction with RNA [27], as with the case of RAVA p4 protein (Table 2). The size of these three proteins was significantly different from other members (Figure 2). Although there was no significant sequence identity with its putative orthologs, the presence of transmembrane domains in p2 and p3 proteins and the NTP binding helicase domain in p4 makes us hypothesize that these proteins constitute the TGB movement proteins of RAVA.

ORFs downstream of the viral replicase are thought to be translated from 3′-terminal subgenomic RNAs in which TGB proteins get translated through leaky scanning mechanisms in all families of the order other than *Tymoviridae* [27]. Sequence context around the initiation codon affects the translation efficiency of mRNAs. The consensus optimal context for translation initiation in mammals is GCC(A/G)CC**AUG**G (the initiation codon in bold; [29]). In some cases, viruses also follow this rule [30]. The Kozak context of translation initiation was observed in RAVA ORF 5, although its initiation codon is 15 nt downstream of the overlapping ORF 4. This is expected if the protein is expressed, given that the ribosomes first identify the start codon of p4, and the CP initiation context needs to be optimal if any ribosomes are to bypass that of p4.

The last ORF of most *Alpha*- and *Betaflexiviridae* members is the CP (Figure 2). However, the last ORF of RAVA lacked the domains conserved in the CP of flexuous viruses [31]. The predicted RNA binding region of the protein is rich in Arg. Arg-rich RNA binding motif in the CP is known to be involved in genome binding and subsequent packaging [32]. The protein also has aa residues thought to be involved in Ca binding. Calcium plays an important role in the assembly and disassembly and/or replication processes in viruses [33,34,35]. The size of the protein is similar to the range shown by members that have TGB MP (28–45 K; Figure 2). The position in the genome, predicted size, Arg-rich RNA binding region, and presence of Ca binding sites suggests that the protein is the RAVA CP.

The genomes of the members of *Tymovirales* are highly diverse with respect to the number and organization of genes, indicative of the major role of recombination in the evolution of this virus group. There is significant diversity in the 3′ region downstream of viral replicase (30 K/TGB MP, CP, and NABP) suggestive of possible recombination events in which the ancestral viruses had a common 5′ part while acquiring the 3′ genes from different origins, which further diversified over time [36]. Several studies have also described evidence of recombination in the members of this group [37,38,39,40,41]. Martelli et al. [26] suggested coevolution of MP and CP in this virus group. RAVA genome follows this theory in which the replicase, although with low bootstrap values, groups with the *Betaflexiviridae*, whereas the remaining proteins are phylogenetically distant. Changes in phylogenetic relationships for different regions of the genome of a virus is an indication of recombination [42]. RAVA polymerase is similar to the members of the *Trivirinae* subfamily, however the rest of the genome is *Quinvirinae*-like (*Carlavirus*, *Foveavirus*, *Robigovirus*; Figure 2) in which the TGBp-like proteins’ organization is reversed, suggesting the role of recombination between these two subfamilies in the evolution of this novel virus.

## 5. Conclusions

RAVA is a unique, previously undescribed virus of tymo-like lineage which cannot be assigned to any of the currently recognized virus taxa. With regard to its molecular features such as the number of genes encoded in the genome, organization, and the presence of conserved pentanucleotide at 5′ termini, RAVA resembles members of the order *Tymovirales*. Phylogenetic analysis based on conserved RdRp domain, the principal determinant in the evolutionary framework of positive-strand RNA viruses, and MTR domain, clearly qualify RAVA as a putative species in the *Betaflexiviridae* family. However, differences in the genome downstream of polymerase (organization of TGBp-like proteins and lack of conserved domains of ortholog proteins) and the distant phylogenetic relationship with other members distinguishes it from both existing genera and unassigned members of the family, suggesting a tentative classification. Therefore, we propose that RAVA represents a new, monotypic genus in the family *Betaflexiviridae* or a family (given the low replicase aa identities with existing viruses) for which the names *Ravavirus*/*Ravaviridae* are proposed. 

## Figures and Tables

**Table 1:** Details of virus isolates used for the phylogenetic studies of Ribes americanum A. RdRp-RNA dependent RNA polymerase, MTR-Methyl transferase, α-*Alphaflexiviridae*, β-*Betaflexiviridae*, γ-*Gammaflexiviridae*, Λ-*Deltaflexiviridae*, T-*Tymoviridae*.

Virus Acronyms	Virus Names	Genus (Family)	RdRp/MTR
ACLSV	Apple chlorotic leaf spot virus	*Trichovirus* (β)	NP040551.1
CMLV	Cherry mottle leaf virus	*Trichovirus* (β)	AOY07780.1
GVA	Grapevine virus A	*Vitivirus* (β)	AFV73358.1
GVB	Grapevine virus B	*Vitivirus* (β)	AIL90366.1
CNRMV	Cherry necrotic rusty mottle virus	*Robigovirus* (β)	NP059937.1
AOPRV	African oil palm ringspot virus	*Robigovirus* (β)	YP002776347.1
CtChV-1	Carrot Ch virus 1	*Chordovirus* (β)	AHA85534.1
CtChV-2	Carrot Ch virus 2	*Chordovirus* (β)	AHA85531.1
DiVA	Diuris virus A	*Divavirus* (β)	YP006905850.1
DiVB	Diuris virus B	*Divavirus* (β)	AFV57240.1
AVCaV	Apricot vein clearing associated virus	*Prunevirus* (β)	AKN09002.1
CPrV	Caucasus prunus virus	*Prunevirus* (β)	AKN08994.1
PVT	Potato virus T	*Tepovirus* (β)	AFU55321.1
PrVT	Prunus virus T	*Tepovirus* (β)	YP009051684.1
ASGV	Apple stem grooving virus	*Capillovirus* (β)	APT42870.1
CVA	Cherry virus A	*Capillovirus* (β)	AMH87272.1
GarCLV	Garlic common latent virus	*Carlavirus* (β)	AGG13282.1
PVM	Potato virus M	*Carlavirus* (β)	AHL30493.1
ASPV	Apple stem pitting virus	*Foveavirus* (β)	NP604464.1
ApLV	Apricot latent virus	*Foveavirus* (β)	YP004089619.1
CLBV	Citrus leaf blotch virus	*Citrivirus* (β)	NP624333.1
BanMMV	Banana mild mosaic virus	Unassigned (β)	NP112029.1
SCSMaV	Sugarcane striate mosaic-associated virus	Unassigned (β)	NP624313.1
BVX	Banana virus X	Unassigned (β)	AAW50958.1
BotV-F	Botrytis virus F	*Mycoflexivirus* (γ)	NP068549.1
ShVX	Shallot virus X	*Allexivirus* (α)	NP620648.1
ICRSV	Indian citrus ringspot virus	*Mandarivirus* (α)	AAK97522.1
PVX	Potato virus X	*Potexvirus* (α)	BAE07083.1
LoLV	Lolium latent virus	*Lolavirus* (α)	YP001718499.1
BVE	Blackberry virus E	Unassigned (α)	AEI17897.1
SaDFV1	Soybean associated deltaflexivirus 1	*Deltaflexivirus* (Λ)	ALM62223.1
FgDFV1	Fusarium deltaflexivirus 1	*Deltaflexivirus* (Λ)	YP009268710.1
SsDFV1	Sclerotinia deltaflexivirus 1	*Deltaflexivirus* (Λ)	AMD16208.1
GFkV	Grapevine fleck virus	*Maculavirus* (T)	NP542612.1
MRFV	Maize rayado fino virus	*Marafivirus* (T)	NP115454.1
TYMV	Turnip yellow mosaic virus	*Tymovirus* (T)	AMH40140.1

**Table 2:** RNA interacting amino acid residues predicted in Ribes americanum virus A proteins. Amino acid residues in red color are predicted to interact with RNA. Shaded regions are predicted transmembrane domain(s) in p2 and p3 proteins whereas in p4 it indicates the region aligned to the helicase domain of ATP-dependent DNA helicase.

**p2**
MNFKSYLLKKIKSVGIGLASSLIIYIASFVFNVLYRRSF
p3
MCYIDVAFDLVCLFICVLILVALLKLTYCNSSAFCVALALTIYSLFLNFNLLVLLYDLSR
p4
MYGYNNGIRKTSDRFSKGSVSKDKYGQRYNCGTDRLPFLVMADVSKLKIDFENATENMLFQIVSLLLHFCVLQNIGQ RKAKRGKIKKKKAAYNEYRRNKDGASSSYQGGGGLARTRDSQENERQVDAARDKRAEFYS
DSSSTEGDGDGSGQTRNERHFV
CP
MESEKLVIVSAKVPFRRTSMAKDTTAARTDFLSSLWRMSLNSKLISKMRQRTCYSRLCHSCCTSAYCKILARGRQREERLRRRKLPIMSTGEIRMEPLPVTREGEAWLELEIARKMKGKLTLQETNGRNSILTVAQPKEMV
MDLDRPEMRDILFNLDFTKRLIDQDVFVCSYLVKKAKRVGVEVCTDFHCYFVDTDMTVSALLDAIEIASFFGCINSAVFEICATGSCLCKVGLRELIIEVEKRTIEIPLKCGYHGIKHLTEVEDRQWKVLCANPLIKLEEIEE IYIFWNSLGLKNHERHVKALLDVNGLKES TLRILGAI

## Supplementary Materials

The following are available online at http://www.mdpi.com/1999-4915/10/8/406/s1, Figure S1: Predicted secondary structures (PSI-Pred; [14]) of the Ribes americanum virus A proteins. Table S1: Primers and PCR conditions used for the amplification of the Ribes americanum virus A genome.
