# Supplementary material for: A Virus in American Blackcurrant (Ribes americanum) with Distinct Genome Features Reshapes Classification in the Tymovirales
Source: Viruses. 2018 Aug 3;10(8):406. doi: 10.3390/v10080406 (PMC6115964; doi:10.3390/v10080406)
Supplement: Supplementary file 1 [file viruses-10-00406-s001.pdf]

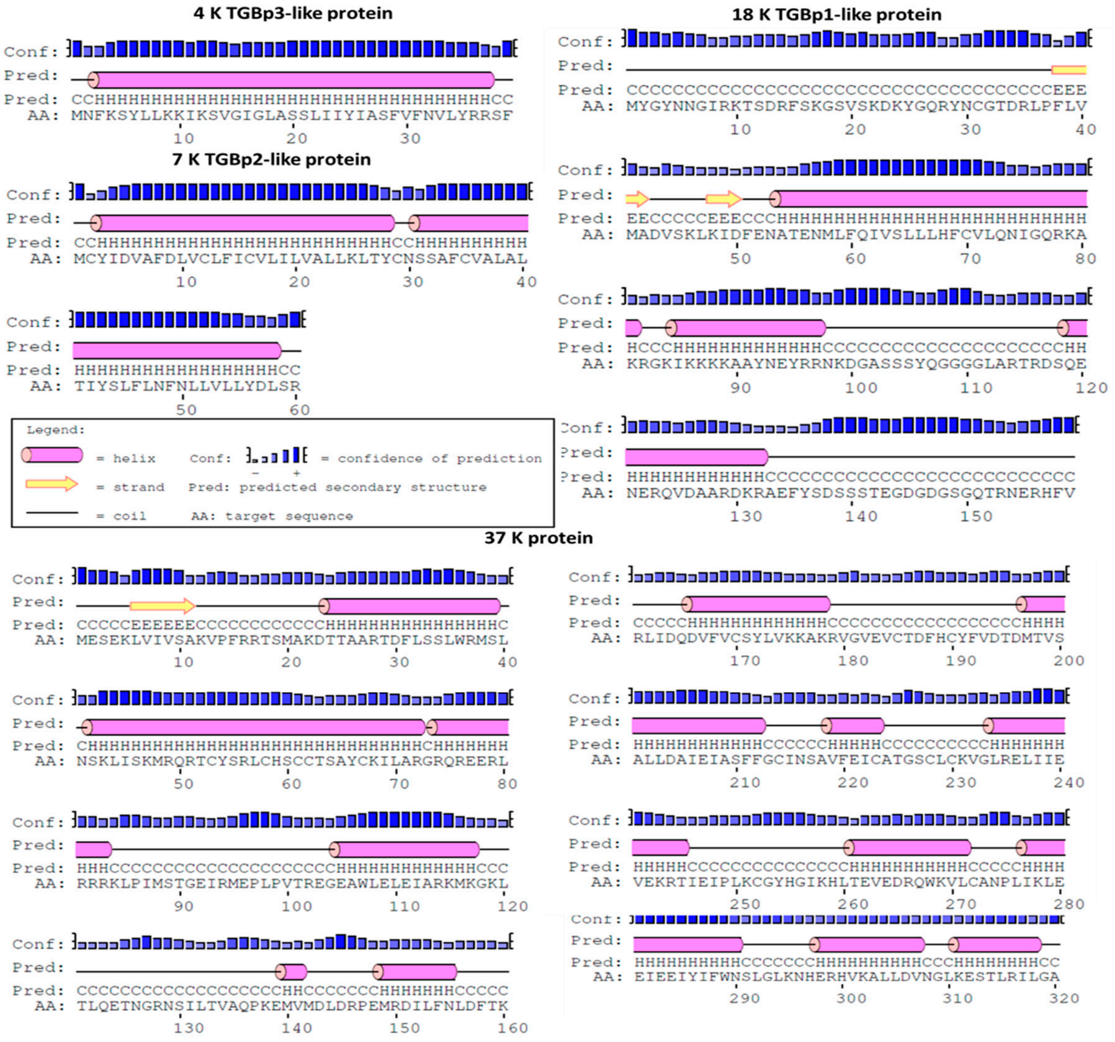

Figure S1. Predicted secondary structures (PSI-Pred; [14]) of the Ribes americanum virus A proteins.

Table S1. Primers and PCR conditions used for the amplification of the Ribes americanum virus A genome.

Primer Name	Primer sequences	Position in the genome	PCR product size (bp)	PCR conditions
For confirmation of HTS assembly				
RAVAORF1F2	(+) 5′-TGAGACACAAGAACATGCTGTTTGA-3′	nt 5,539-5,563	979	Initial denaturation at 94°C for 2 min followed by 40 cycles of 94°C-20 s, 54°C-20 s and 72°C-1 min followed by 5 min final extension at 72°C.
RAVAORF5R1	(-) 5′-TTCCACCCCTACTCTCTTTGCCT-3′	nt 6,495-6,517		
RAVAORF1F3	(+) 5′-TCTGAAGGGTCTGCGTGACAGCTT-3′	nt 5,570-5,593	948	
RAVAORF5R1	(-) 5′-TTCCACCCCTACTCTCTTTGCCT-3′	nt 6,495-6,517		
RAVAORF1F1	(+) 5′-TTGAAGGAAGTGATCAACAGCTA-3′	nt 5,415-5,437	681	Initial denaturation at 94°C for 2 min followed by 40 cycles of 94°C-20 s, 52°C-20 s and 72°C-45 s.
RAVAORF4R1	(-) 5′-TTGAGTTTAGAGACATCCGCCAT-3′	nt- 6,073-6,095		
RAVAORF4F1	(+) 5′-AACTAGTGATCGTTTCAGCAAAGGT-3′	nt 5,982-6,006	636	
RAVAORF5R2	(-) 5′-AATTGATACATCCGAAGAAACTT-3′	nt 6,595-6,617		
For 5′ and 3′ RACE				Initial denaturation at 94°C for 2 min followed by 40 cycles of 94°C-20 s, 64°C-20 s and 72°C-1 min.
RLM 5′ RACE outer primer	(+) 5′-GCTGATGGCGATGAATGAACACTG-3′			
RAVAORF1R1	(-) 5′-AACTGGCCATATCAAAAACCGTT-3′	nt 560-582	627	
RLM 5′ RACE outer primer	(+) 5′-GCTGATGGCGATGAATGAACACTG-3′			Initial denaturation at 94°C for 2 min followed by 40 cycles of 94°C-20 s, 58°C-20 s and 72°C-1 min.
RAVAORF1R2	(-) 5′-TAGAAGTTCTCAGGAGAGACAGA-3′	nt 678-700	745	
PCR- oligo(dt) primer	(-) 5′-GGCCACGCGTCGACTAGTAC(T18)-3′			
RAVAORF1F2	(+) 5′-TGAGACACAAGAACATGCTGTTTGA-3′	nt 5,539-5,563	1606	
PCR- oligo(dt) primer	(-) 5′-GGCCACGCGTCGACTAGTAC(T18)-3′			Initial denaturation at 94°C for 2 min followed by 40 cycles of 94°C-20 s, 51-58°C-20 s and 72°C-2 min 30 s followed by 5 min final extension at 72°C.
RAVAORF1F3	(+) 5′-TCTGAAGGGTCTGCGTGACAGCTT-3′	nt 5,570-5,593	1575	
PCR- oligo(dt) primer	(-) 5′-GGCCACGCGTCGACTAGTAC(T18)-3′			
RAVAORF5F1	(+) 5′-TGAAAGGCAAGTTGATGCTACA-3′	nt 6,315-6,336	830	
Reverse transcription primers				
RAVA5′R1	(-)5′-TTGAGGTCTTGAACTTATTGGCCTTA-3′	nt 860-885		
RAVA5′R2	(-)5′-ATCTCACTATGTACCCCATCCGGTGA-3′	nt 801-826		
RT- oligo(dt) primer	(-) 5′-CTACTACTACTAGGCCACGCGTCGACTAGTAC(T18)-3′			

(+)- forward primer, (-)- reverse primer.
